# Danggui Sini Decoction Protected Islet Endothelial Cell Survival from Hypoxic Damage via PI3K/Akt/eNOS Pathway

**DOI:** 10.1155/2018/5421023

**Published:** 2018-07-10

**Authors:** Wenting Chen, Caoxin Huang, Chen Yang, Xilin Ge, Wenfang Huang, Xuejun Li, Shuyu Yang, Suhuan Liu

**Affiliations:** ^1^Xiamen Diabetes Institute, The First Affiliated Hospital of Xiamen University, Xiamen 361003, China; ^2^Medical College of Xiamen University, Xiamen 361000, China; ^3^Central Laboratory, The First Affiliated Hospital of Xiamen University, Xiamen 361003, China

## Abstract

Danggui Sini decoction (DSD) is a traditional Chinese decoction, which is wildly applied and showed to be effective in ameliorating ischemia-related symptoms. However, the mechanisms of DSD action in ischemic damage remain to be fully clarified. Pancreatic islet endothelial cells are pivotal constituent of islet microvasculature, with high vulnerability to hypoxic injuries. Here, using MST1 cell, a pancreatic islet endothelial cell-line, as a model, we investigated the effects of DSD on hypoxia-stimulated endothelial cell lesions and its underlying mechanisms. We found that DSD-Containing Serum (DSD-CS), collected from DSD-treated rats, could efficiently protect MST1 survival and proliferation from Cobalt chloride (CoCl_2_) induced damage, including cell viability, proliferation, and tube formation. Furthermore, DSD-CS restored the activity of PI3K/Akt/eNOS signaling inhibited by CoCl_2_ in MST1 cells. The protective effect of DSD-CS could be blocked by the specific PI3K/Akt/eNOS inhibitor LY294002, suggesting that DSD-CS protection of MST1 cell survival from hypoxia was mediated by PI3K/Akt/eNOS pathway. In conclusion, DSD treatment protected MST1 survival from hypoxic injuries via PI3K/Akt/eNOS pathway, indicating its role in protecting microvascular endothelial cells.

## 1. Introduction

Danggui Sini decoction (DSD), first reported in* Shanghan Lun*, is a commonly used traditional Chinese medicine in increasing cardiovascular and peripheral circulation [1]. In addition, DSD is also applied in treating watery diarrhea, shock, heart failure, and severe poor extremity circulation [2–5]. It is extracted from* Angelica sinensis*, Ramulus Cinnamomi, and Radix Puerariae for nourishing blood, antagonizing vascular diseases, and hemodynamic instability [2, 5, 6]. Angelical sinensis acts as a traditional phytochemicals, which was first reported in* Shennong Bencao Jing*, with ability to promote angiogenesis [1, 3, 5, 7]. Ramulus Cinnamomi is a principal bioactive ingredient with antioxidant and anti-inflammatory effects [8–12]. Puerarin is a traditional Chinese herbal, which shows a great effect in protecting islets cells from oxidative stress by activating antioxidant enzymes [13, 14]. In previous study, we have reported that DSD was effective in ameliorating diabetic peripheral neuropathy (DPN), one of the common and typical diabetes microvascular complications [6].

Microvascular endothelial cells form a physiologically vital interface between the circulating blood and surrounding microenvironment and thus play a key role in regulating the access of cells and blood molecules into the tissue under a variety of conditions including inflammation, repair, and survival [15, 16]. Besides, uninterrupted blood flow through the microvasculature is critical for overall organ function to supporting nutrition and adequate oxygen [15–18]. As the typical component of islet microvasculature and microcirculation [19], pancreatic islet endothelial cells are critical for oxygen and hormones transportation and, therefore, play important roles in the pancreas *β*-cell survival and proliferation [20–22], with high vulnerability to hypoxic injuries [23–26]. Thus the pancreatic islet endothelial cell may serve as a good model for studying microvascular endothelial cell biological activities.

Here, using a pancreatic islet endothelial cell-line MST1 cell as a microvasculature model, we designed the present study to evaluate whether Danggui Sini decoction can protect endothelial cells function and survival against hypoxic stress* in vitro*.

## 2. Materials and Methods

### 2.1. Materials

Cobalt chloride (CoCl_2_), a chemical to induce hypoxia-like reactions, was purchased from Sigma-Aldrich (Merck Millipore, Darmstadt, Germany). Cell Counting Kit-8 (CCK-8) was obtained from Dojindo Laboratories (Kumamoto, Japan). In Situ Cell Death Detection Kit for TUNEL assay was obtained from Roche (Basel, Switzerland). Cell-Light™ EdU Apollo488 In Vitro Kit was obtained from Ribobio (Guangzhou, China). Matrigel Matrix Growth Factor Reduced (356230) was purchased from Corning Incorporated (Tewksbury, MA, USA). Akt, p-Akt, p-eNOS, eNOS, and *β*-actin antibodies were purchased from Cell Signaling Technology (Boston, MA, USA). LY294002, a PI3K inhibitor, was obtained from Cell Signaling Technology (Boston, MA, USA).

### 2.2. Preparation of DSD-Containing Serum (DSD-CS)

The DSD was prepared by the Department of Pharmacy of the First Affiliated Hospital of Xiamen University, China. Male Sprague-Dawley rats (350-400 g) were purchased from Shanghai SLAC Laboratory Animal Co. Ltd. (Shanghai, China). All procedures conducted in the animal experiments were approved by Xiamen University Animal Care and Use Committee. Rats were divided into two groups randomly. One group was given DSD (100 mg/kg, dissolved in water) with daily gavage for 7 consecutive days, while the other group was given an equal volume of water only. On day 7, 2 hours after the last oral administration of DSD, rats were anesthetized, and the blood was collected from abdominal aorta and kept at 4°C overnight. The second day, blood was centrifuged at 3000 rpm for 10 min and the serum was collected. The serum was inactivated for 30 mins at 56°C and then kept at -20°C until use.

### 2.3. Quality Control for DSD and DSD-Containing Serum (DSD-CS)

**Figure 1:**
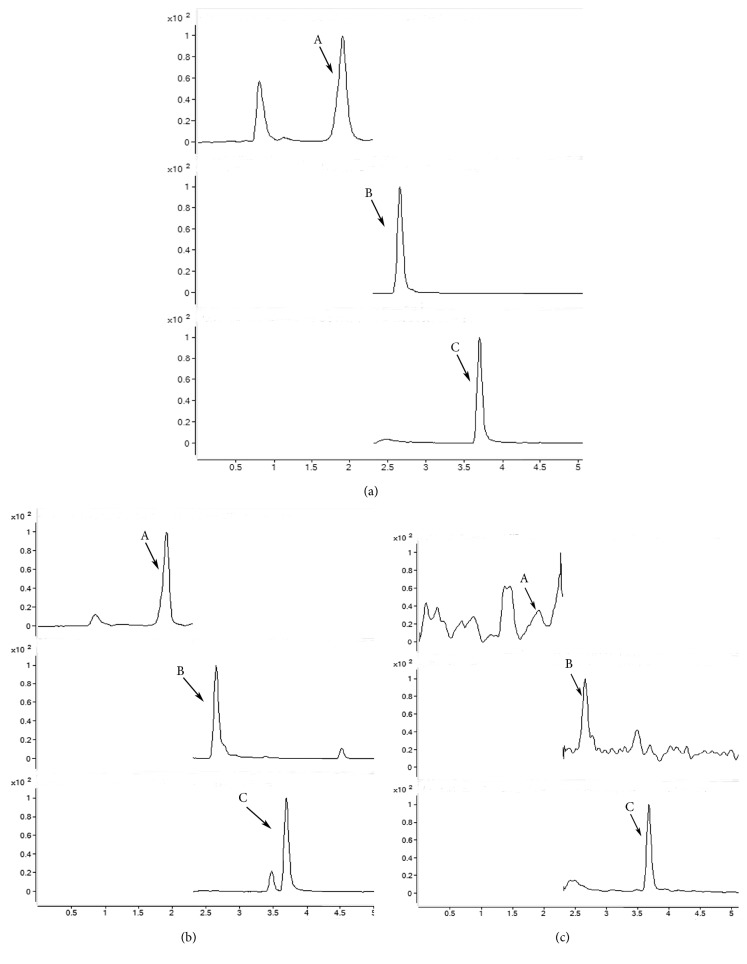
Mass spectrometer HPLC analysis of the reference standards (a), DSD (b), and DSD-Containing Serum (DSD-CS) (c). The peaks corresponding to puerarin (A), ferulic acid (B), and cinnamic acid (C) were identified.

As described in the method and material part, we have compared the chemical composition of DSD and DSD-Containing Serum using mass spectrometer HPLC analysis (Figure 1). The chemical compositions of DSD itself and DSD-Containing Serum (DSD-CS) were determined by an Agilent 6410 triple stage quadrupole mass spectrometer equipped with an ESI ion source and an Agilent 1290 HPLC system with autosampler (Agilent Technologies, Santa Clara, CA, USA). The analytes were separated on ACQUITY UPLC HSS C18 (2.1 × 100 mm, 1.8 *μ*m) used at 40°C. The mobile phase was used with a gradient elution: 0–5 min, 10-80 % B, and 5-7 min, 80-90 %, at a flow rate of 0.3 ml/min. ESI-MS/MS conditions were set as follows: gas temperature 300°C, gas flow 5 l/min, capillary: positive 4000V, negative 3500V, and nebulizer pressure 45 psi. MS acquisition was performed in multiple reaction monitoring (MRM) mode. The compound dependent parameters used for analysis were summarized in Table 1. The result was shown in Figure 1. By comparing to reference standards, major peaks were identified as ferulic acid, cinnamic acid, and puerarin. DSD is the same as DSD-Containing Serum (DSD-CS).

### 2.4. Cell Culture and Treatment

MST1 cell, an islet endothelial cell-line, was purchased from American Type Culture Collection (ATCC). MST1 were cultured in RPMI 1640 medium supplemented with 10% fetal bovine serum (FBS), streptomycin (100 *μ*g/mL), and penicillin (100 Units/mL). Cells were exposed to CoCl_2_ with or without DSD-CS (10%, v/v) for 12 hours or 24 hours. According to the results of dose and time course tests, CoCl_2_ stimulation at 200 *μ*M for 24 hours was applied in the rest of the study.

### 2.5. Cell Viability Assay

Cell viability was evaluated by Cell Counting Kit. MST1 cells were seeded in 96-well plates (5000 cells/ well) and exposed to CoCl_2_ (200 *μ*M) with or without DSD-CS for 24 hours. Then 10 *μ*L CCK-8 reagent was added to each well of the plates and incubated at 37°C for 1 hour. The absorbance was measured with the Bio-Tek Synergy H1 Microplate Reader (Winooski, VT, USA) at 450 nm.

### 2.6. Apoptosis Assay

Cell apoptosis was evaluated by In Situ Cell Death Detection Kit with counterstaining by DAPI. Images were taken by a fluorescence microscope (Olympus, Shanghai, China).

### 2.7. Cell Proliferation Assay

Cell proliferation was evaluated by Cell-Light™ EdU Apollo488 In Vitro Kit. Briefly, MST1 cells were fixed in the 4% paraformaldehyde after treatment at room temperature for 30 min and then stained with Apollo® for 30 min. Nuclei were stained with Hoechst 33342 at room temperature for 30 min. Images were taken by a fluorescence microscope (Olympus, Shanghai, China).

### 2.8. Tube Formation Assay

Tube formation capacity was measured by Matrigel tube formation assay. Following the treatment, MST1 cells (5×10^4^ cells) were counted and seeded on 96-well culture plates precoated with 50*μ*l of Matrigel Matrix at 37°C for 30 minutes in advance. Images were taken using the fluorescent microscope 1 hour after seeding.

### 2.9. Western Blot Analysis

The MST1 cells were collected and lysed for protein extraction. Protein (25 *μ*g) was separated by 10% polyacrylamide gels and transferred to the nitrocellulose membrane. The membranes were blocked by 5% BSA in PBST and incubated with primary antibodies overnight at 4°C followed by secondary antibody incubation at 1:5000 dilution for 1 hour at room temperature. The blots were developed in chemiluminescence (ECL) system and Kodak X-OMAT film.

### 2.10. Statistical Analysis

Results were presented as mean ± SEM from three independent experiments at least. GraphPad Prism 5.0 software (GraphPad, CA, USA) was applied for data analysis. Statistical analysis among different groups was carried out with the paired* t*-test.* P *< 0.05 was considered statistically significant.

## 3. Results

### 3.1. Cobalt Chloride (CoCl_2_) Dose-Dependently Reduced MST1 Survival and Proliferation

**Figure 2:**
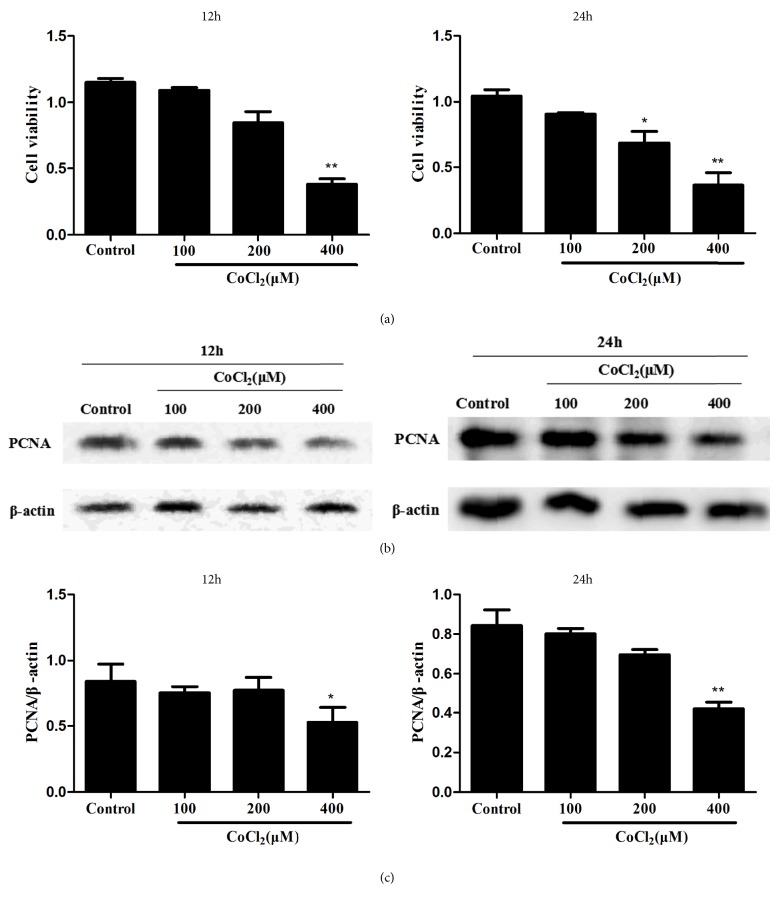
MST1 survival and proliferation were dose-dependently reduced by CoCl_2_ treatment. The cells were treated with CoCl_2_ ranging from 100 to 400 *μ*M for 12 hours or 24 hours and measured with CCK8 assay (a) and for PNCA protein level (b, c). ∗*P* < 0.05 and ∗∗*P* < 0.01 versus Control group.

To investigate how MST1 respond to hypoxia stimuli, CoCl_2_ was applied in this study to mimic hypoxia status. As was shown in Figure 2, CoCl_2_ could damage cell viability dose-dependently as measured in Cell Counting Kit-8 (CCK8) assay. PCNA (proliferating cell nuclear antigen), identified as a marker of DNA synthesis during cell cycling, also decreased upon hypoxia stimulation indicating that MST1 proliferation was inhibited. These results suggested that CoCl_2_ could inhibit MST1 cell survival and proliferation capacity. CoCl_2_ stimulation at 200 *μ*M for 24 hours was determined for the rest of this study.

### 3.2. Cobalt Chloride (CoCl_2_) Dose-Dependently Inhibited Phosphorylation of PI3K/Akt and eNOS

**Figure 3:**
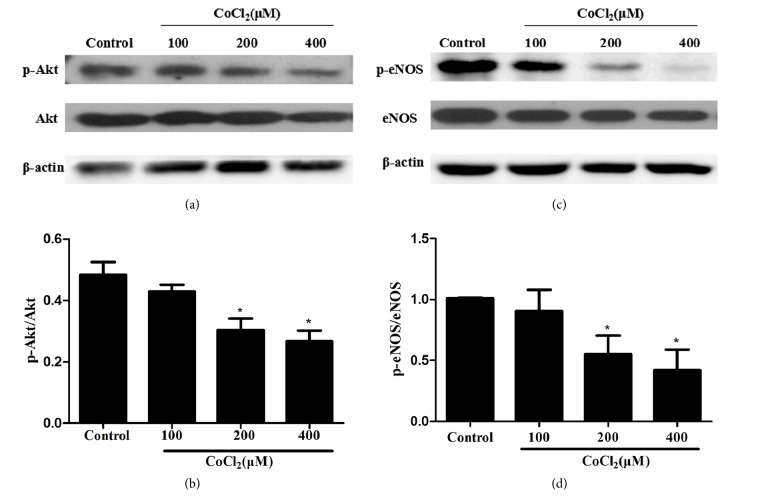
Phosphorylation of PI3K/Akt and eNOS was dose-dependently inhibited by CoCl_2_ treatment. The cells were treated with different concentrations of CoCl_2_ for 12 hours or 24 hours. Protein level of p-Akt was reduced in MST1 cells stimulated with CoCl_2_ (a, b). Protein level of p-eNOS was reduced in MST1 cells stimulated with CoCl_2_ (c, d). ∗*P *< 0.05 versus control group.

PI3K/Akt/eNOS signaling plays a pivotal role in endothelial cell biological functions [27–30]. Here we measured p-Akt, Akt, p-eNOS, and eNOS expression by Western blot. As is shown in Figure 3, CoCl_2_ could reduce p-Akt and p-eNOS. The result suggested that PI3K/Akt/eNOS signaling pathway was correlated to CoCl_2_ induced MST1 dysfunction.

### 3.3. DSD-CS Protected Cell Survival and Proliferation from Hypoxia-Induced Damage is PI3K/Akt Dependent

**Figure 4:**
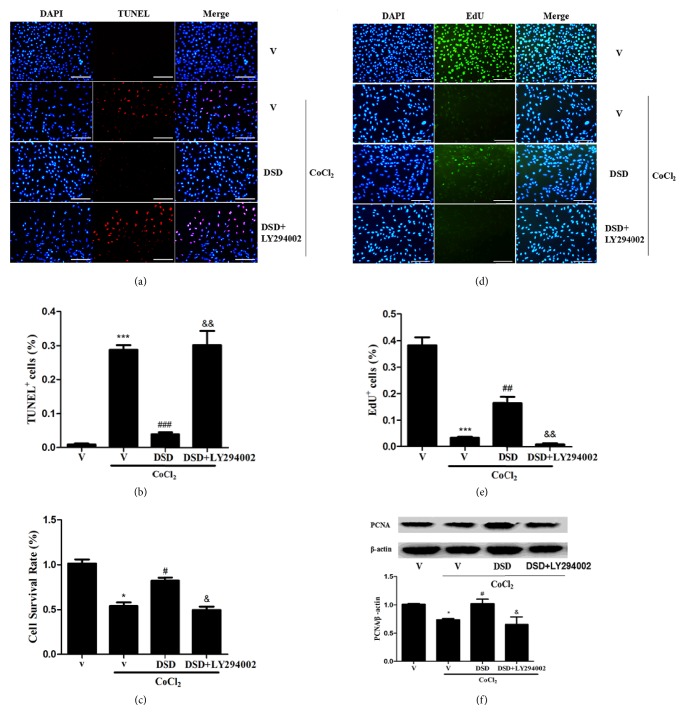
Cell survival and proliferation were protected by DSD-CS from hypoxia-induced damage. (a-c) The cell survival capacity was measured by TUNEL assay and CCK8 assay. ∗*P *< 0.05 and ∗∗∗*P *< 0.001 versus vehicle group (V); ^#^*P *< 0.05 and ^###^*P* < 0.001 versus CoCl_2_ + vehicle group (V); ^&^*P *< 0.05 and ^&&^*P* < 0.01 versus CoCl_2_ + DSD group. (d-f) The cell proliferation was measured by EdU assay and PCNA expression. ∗*P *< 0.05 and ∗∗∗*P* < 0.001 versus vehicle group (V); ^#^*P *< 0.05 and ^##^*P* < 0.01 versus CoCl_2_ + vehicle group (V); ^&^*P *< 0.05 and ^&&^*P* < 0.01 versus CoCl_2_ + DSD group. Scale bars: 200 *μ*m.

To determine whether DSD-CS could protect cell survival from hypoxia-induced damage, CCK8 and TUNEL assay were applied in this study. As presented in Figure 4, exposure to 200 *μ*M CoCl_2_ for 24 hours significantly reduced cell survival compared with the vehicle group. The decrease of cell survival was abrogated by DSD-CS treatment, which was attenuated by PI3K/Akt inhibitor LY294002. Furthermore, we measured cell proliferation with Cell-Light™ EdU assay and PCNA expression. As shown in Figure 4, compared with the vehicle group, CoCl_2_ treatment significantly reduced cell proliferation, which was partially restored by DSD-CS. The reversed cell proliferation by DSD-CS was attenuated by LY294002. These results suggested that DSD-CS protected cell survival and proliferation from hypoxia-induced damage, in which PI3K/Akt signaling was involved.

### 3.4. DSD-CS Protected MST1 Tube Formation Capacity from Hypoxia-Induced Damage Was PI3K/Akt Dependent

**Figure 5:**
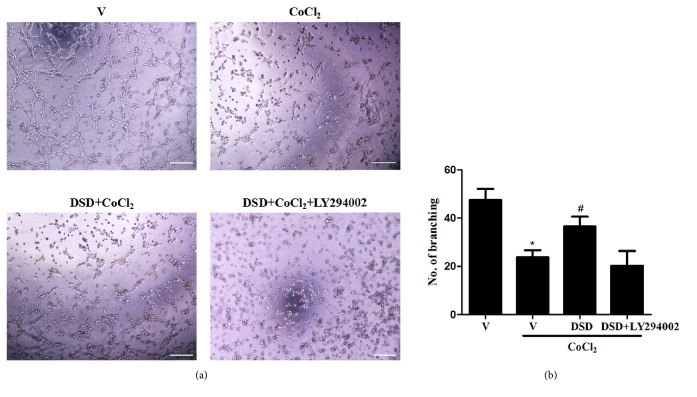
DSD-CS protected MST1 tube formation capacity from hypoxia-induced damage and was PI3K/Akt dependent. (a) The cells tube formation was measured by Matrigel tube formation assay. (b) Number of branching was quantified. ∗*P *< 0.05 versus vehicle group (V); ^#^*P *< 0.05 versus CoCl_2_ + vehicle group (V). Scale bars: 200 *μ*m.

A Matrigel tube formation assay was performed to measure the morphology and tube formation capacity of MST1. Compared to the vehicle group, CoCl_2_ treatment significantly reduced the amount of tubes formation, which was antagonized by DSD-CS. The reversed tube formation by DSD-CS was blocked by PI3K/Akt inhibitor LY294002, suggesting that PI3K/Akt signaling is involved in DSD-CS mediated improvement of cell tube formation capacity (Figure 5).

### 3.5. DSD-CS Mediated Protective Action Activated PI3K/Akt/eNOS Signaling

**Figure 6:**
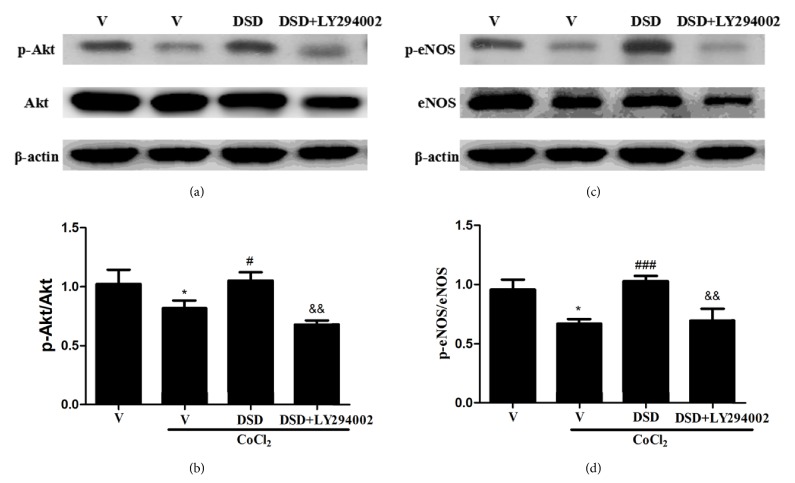
Effects of DSD on PI3K/Akt/eNOS signaling in CoCl_2_ treated MST1. (a-b) Representative Western blots of total and phosphorylated Akt protein expression. ∗*P *< 0.05 versus vehicle group (V); ^#^*P *< 0.05 and ^###^*P* < 0.001 versus CoCl_2_ + vehicle group (V); ^&^*P *< 0.05 and ^&&^*P* < 0.01 versus CoCl_2_ + DSD group; (c-d) Representative Western blots of total and phosphorylated eNOS protein expression. ∗*P *< 0.05 versus vehicle group (V); ^#^*P *< 0.05 and ^###^*P* < 0.001 versus CoCl_2_ + vehicle group (V); ^&^*P *< 0.05 and ^&&^*P* < 0.01 versus CoCl_2_ + DSD group.

To investigate whether the effect of DSD-CS was mediated by PI3K/Akt/eNOS signaling pathway, we measured p-Akt, Akt, p-eNOS, and eNOS expression by Western blot. As shown in Figure 6, hypoxia stimulation reduced level of p-Akt compared to the control group. DSD-CS treatment can partially antagonize the reduction. The effect was blocked by LY294002. Meanwhile, we found that CoCl_2_ reduced p-eNOS protein level in MST1 after 24 hours of incubation, which was abrogated by DSD-CS. Similarly, LY294002 could also block the reverse effect of DSD-CS indicating the involvement of Akt pathway in DSD-CS effect.

## 4. Discussion

Danggui Sini decoction (DSD) is a commonly used Chinese traditional medicine in increasing cardiovascular and peripheral circulation [1]. Previous researches had demonstrated that it is effective in treating vascular diseases, including Raynaud's phenomenon (RP), shock, heart failure, and severe poor extremity circulation [1–5]. Furthermore, its major components including* Angelica sinensis*, Ramulus Cinnamomi, and Radix Puerariae have been shown to possess great potential in promoting angiogenesis [1, 3, 5, 7] and activating antioxidant enzymes and anti-inflammation [8–14]. Here we showed that DSD greatly protected endothelial cell survival, proliferation, and function under hypoxic conditions.

Mounting studies have revealed the underlying mechanisms of Danggui Sini decoction (DSD) in regulating microcirculation. It was demonstrated that DSD could regulate the lipid metabolism, energy, and amino acid to adjust the fiber protease, platelet aggregation, and the expression of tissue factor [31]. In addition, DSD could alleviate diabetes-induced neuropathic pain by suppressing inflammatory process and gliosis in spinal cord [6]. Pancreas islet has a plenty of microvasculature transporting oxygen and hormones, which plays pivotal roles in supporting and regulating the proliferation and survival of pancreas *β*-cells [20–22]. It may also influence pancreatic islet transplantation, the responsive capacity to insulin resistance of *β*-cells, and the overall islet microenvironmental homeostasis [32, 33]. Our present study demonstrated that DSD could efficiently protect islet derived endothelial cell-line-MST1 against Cobalt chloride (CoCl_2_) induced lesions, including cell viability and proliferation and tube formation.

Endothelial cell viability, proliferation and tube formation capacity could be regulated by mitotic spindle dynamics [34–36], the activation of proapoptotic factors, and the inhibition of antiapoptotic factors [37–39]. PI3K/Akt is reported to be a key signaling pathway involved in regulating endothelial cells viability, proliferation, and angiogenesis [40, 41]. One of its downstream targets is eNOS, which is also a critical regulator in endothelial cell survival and proliferation. By using MST1 cells, the phosphorylation of eNOS was completely blocked in the presence of LY294002 (a specific PI3K/Akt inhibitor). Besides, our study discovered that DSD-CS could reverse the CoCl_2_-induced PI3K/Akt/eNOS inhibition, which was blocked by the specific PI3K/Akt inhibitor LY294002, suggesting that DSD-CS protection of MST1 cell survival from hypoxia was mediated by PI3K/Akt/eNOS pathway.

Lining in the inner surface of blood microvessels, the vascular endothelium acts as the first interface for circulating blood components and regulates the perfusion and blood delivery. Besides, it interacts with extravascular tissues as well as other cell types lining along vasculatures. As a semipermeable barrier, it controls blood–tissue exchange of oxygen, nutrients, and hormones. Upon barrier dysfunction, it will lead to multiple organ dysfunction including metabolic disorder, infection, trauma, and other kinds of disease [42]. Our study demonstrated that DSD-CS treatment could efficiently protect MST1 against hypoxic injuries by maintaining cell viability and proliferation and tube formation capacity indicating that DSD treatment had a direct protection effect on islet endothelial cell.

In summary, we revealed that DSD is effective in protecting the islet endothelial cells from hypoxia-induced damage. DSD protection of endothelial cell survival and proliferation and tube formation capacity involved PI3K/Akt/eNOS pathway. In addition to its multiple roles in treating ischemia-related diseases [1–6], DSD was showed here that it had a direct and potent effect in protecting cultured islet endothelial cell under hypoxic conditions, implying a further application in ameliorating islet microvasculature and microcirculation dysfunction when the hypoxia is present.

## Figures and Tables

**Table 1:** Parameters of ferulic acid, cinnamic acid, and puerarin for MS condition.

Compound Name	Precursor Ion	Product Ion	Fragmentor	Collision Energy	Cell Accelerator Voltage	Polarity
Ferulic acid	193.2	134.2	91	11	0	Negative

Cinnamic acid	147.2	103.2	83	5	7	Negative

Puerarin	417.1	297.1	152	22	0	Positive

## Data Availability

The data used to support the findings of this study are available from the corresponding author upon request.
